# Nursing, history, and orthopedics in manuals (1875-1928)

**DOI:** 10.1590/0034-7167-2022-0567

**Published:** 2023-10-06

**Authors:** Bruna Moura Oliveira dos Santos, Claudia Labriola, Sarah Goes Barreto da Silva Moreira, Hugo Alberto Neves de Souza, Fernando Porto

**Affiliations:** I Universidade Federal do Estado do Rio de Janeiro. Rio de Janeiro, Rio de Janeiro, Brazil

**Keywords:** Nursing, History of Nursing, Orthopedics, History, Nursing Care, Enfermería, Historia de la Enfermería, Ortopedia, Historia, Atención de Enfermería, Enfermagem, História da Enfermagem, Ortopedia, História, Cuidados de Enfermagem

## Abstract

**Objectives:**

to discuss the content of manuals, with emphasis on orthopedics, in support of the development of nursing care culture.

**Methods:**

cultural-historical method articulated with document analysis technique. The sources were nursing manuals - Portuguese, French, English, and Spanish - from 1875 to 1928.

**Results:**

this study pointed to 12 works - 6 authored by physicians, 2 by nurses, 3 institutional, and 1 by a Sister of Charity - that presented, in a transversal way, the professionalization process initiated in Europe. The manuals addressed first aid care and immobilization methods, from the simplest, such as improvised splints, to the application of plaster casts.

**Conclusions:**

the nurses’ work, even in a limited capacity, showed that they were able to observe warning signs so that doctors could act, with some exceptions.

## RESUMO

**Objetivos:**

discutir os conteúdos dos manuais, com ênfase na ortopedia, em prol do desenvolvimento da cultura dos cuidados de enfermagem.

**Métodos:**

método histórico-cultural articulado à técnica de análise documental. As fontes foram os manuais de enfermagem - portugueses, franceses, ingleses e espanhóis -, no período de 1875 a 1928.

**Resultados:**

este estudo apontou para 12 obras - 6 de autoria de médicos, 2 de enfermeiras, 3 institucionais e 1 Irmã de Caridade - que apresentaram, de forma transversal, o processo de profissionalização iniciado na Europa. Os manuais abordaram cuidados nos primeiros socorros e maneiras de imobilização, desde as mais simples, como as talas improvisadas, até a aplicação do aparelho gessado.

**Conclusões:**

a atuação das enfermeiras, mesmo que de forma limitada, mostrava que elas eram capazes de observar os sinais de alerta para que os médicos pudessem atuar, salvo algumas exceções.

## RESUMEN

**Objetivos:**

discutir los contenidos de los manuales, con énfasis en la ortopedia, en beneficio del desarrollo de la cultura de cuidados de enfermería.

**Métodos:**

método histórico-cultural articulado a la técnica de análisis documental. Las fuentes fueron los manuales de enfermería - portugueses, franceses, ingleses y españoles -, en el período de 1875 a 1928.

**Resultados:**

este estudio señaló 12 obras - 6 de autoría de médicos, 2 de enfermeras, 3 institucionales y 1 de una Hermana de la Caridad - que presentaron, de manera transversal, el proceso de profesionalización iniciado en Europa. Los manuales abordaron cuidados en primeros auxilios y formas de inmovilización, desde las más simples, como las tablillas improvisadas, hasta la aplicación del aparato de yeso.

**Conclusiones:**

la actuación de las enfermeras, aunque limitada, demostraba que eran capaces de observar las señales de alerta para que los médicos pudieran actuar, exceptuando algunas situaciones.

## INTRODUCTION

In the second half of the 19th century, scientific, technological, sanitary, and cultural transformations took place in Europe, specifically in England and France, due to war conflicts and revolutions. These events influenced the teaching of nursing, such as the modern nursing movement led by Florence Nightingale, who founded the School of Nursing at St. Thomas Hospital in London (1860), after the Crimean War (1853-1856). In France, the French Revolution (1789-1799) marked the end of the monarchy and the beginning of the republic with the principles of liberty, equality, and fraternity. The cultural, economic, and political aspects influenced education, especially in nursing schools, in favor of professionalization to replace the Sisters of Charity with the installation of the Third Republic (1870-1940). We highlight the School of Training for Nurses of the Public Assistance, at the Salpêtrière Hospital, led by Désiré-Magloire Bourneville and criticized by Léonie Chaptal, who affirmed its political intentions^(1)^. Bourneville retired in 1905 and did not accept the low attendance for the training of lay nurses, but the institution gained new directions in 1907 and was reconfigured in 1908^(2)^ to the model recommended by Florence after clashes with Chaptal and Anna Hamilton^(1)^.

In the Americas, there were nursing schools prior to the system recommended by Florence. For example, the New England Hospital (1860), located in Boston/Massachusetts, with the exemplary adoption of the deaconesses of Kaiserswerth^(3)^. In South America, we had in Argentina the Municipal School of Nurses Dr. Cecilia Grierson, in Buenos Aires (1890), under the influence of Florence’s model^(4)^, and in Brazil, the Professional School of Nurses (1890), now the Alfredo Pinto School of Nursing, strongly influenced by the French model of Désiré-Magloire Bourneville. Years later, the School of Nurses of the National Department of Public Health (1923), now the Anna Nery School of Nursing, was created following the North American model.

In the 1910s, both sides of the ocean suffered from World War I (1914-1918), with due proportions, and as a consequence, the Spanish flu. Despite the name referring to Spain, its origin should be considered in the United States of America, where the first signs occurred, since the country maintained a neutral position in the war conflict^(5)^.

The 1920s marked both sides of the ocean, however, we highlight one event: the New York Stock Exchange Crash (1929) and its worldwide effects^(6)^. This occurred due to the greater quantity of stocks than investors, causing the economy to plummet brutally, as a result of financial speculation and overproduction in the industry. The effect was worldwide economic recession.

In this context, the Rockefeller Foundation, which promoted modern nursing in Brazil and other areas, reduced its investment, causing obstacles in development^(7)^. However, the School of Nurses of the National Department of Health realigned itself, and the result was the promulgation of Decree No. 20,109/1931, when it became the official standard of nursing education in the country.

In summary, as we had the opportunity to show, there were sociocultural, political, economic, and health movements in various continents. In this sense, we believe that several manuals were written in teamwork^(7)^. In this line of thought, manuals written by authors, in addition to recording conduct to be executed by nurses, left cultural clues in the way of caring, based on the accumulated experiences in war conflicts. Historically, nursing ascends in the context of wars^(8)^, as well as in public calamities and reforms. To this end, we cite the participation of some women and nurses. For example, in England, Florence Nightingale (1820-1910), whom we believe requires no further introduction from the previous statements; in France, Léonie Chaptal (1873-1937), a nurse with the Aptitude Certificate École de la Pitié (1903), worked in Paris and participated in the creation of the School of Nurses’ House (1905), serving as director until 1909^(1)^. In Spain, Concepción Arenal (1820-1893), a writer and lawyer, carried out a project in favor of the Spanish reform for the reconfiguration of the hospital system, with field studies in hospitals, especially in Madrid, but without the expected success due to the country’s instability^(9)^.

In the Americas, particularly in the United States of America, Clara Barton (1821-1912), a nurse who served in the Civil War (1861-1865), was one of the creators of the American Red Cross (1881)^(10)^. In Argentina, we highlight Maria dos Remedios del Valle (1766/67-1847), known as the “Mother of the Nation”, an Afro-Argentine who joined the Auxiliary Army with her family to fight for Argentine independence in the Northern provinces (1810-1818). Despite lacking specific training, she provided care for the wounded in the conflict, and upon returning alone without her family, was recognized as a nursing pioneer for her demonstration of love, patriotism, and sacrifice to future generations of nurses^(11)^. In Brazil, Anna Justina Ferreira Nery (1814-1880), a widow from Bahia known by the pseudonym “Mother of Brazilians,” volunteered in the Paraguayan War (1864-1870), where she provided care for the wounded and sick. Recognized as the first Brazilian nurse due to the honors she received upon returning from the war^(12)^.

These experiences, especially in the care of war casualties, influenced the development of a culture of care, particularly for soldiers with orthopedic injuries resulting in bone trauma that affected their mobility. The care required for bone trauma was included in manuals as a means of transmitting and communicating information and modes of action. This diversity of approaches influenced the development of nursing care and was shaped by historical and demographic factors, which form the foundation of a culture focused on health, illness, and the realities of healthcare^(13)^. Therefore, we understand that these factors guided the culturalization of orthopedic nursing care.

It is worth noting that the term “orthopédie” originated from the French language and is derived from the Greek words “orthos” meaning straight, right, and “paidós” meaning child. It was created in 1741 by the French physician Nicolas Andry in his book titled Orthopédie or the Art of Preventing and Correcting Deformities in Children^(14)^.

Thus, the object of study is the culture of orthopedic care through nursing manuals as a means of educating nurses.

## OBJECTIVES

To discuss the contents of nursing manuals, with emphasis on orthopedics, for the development of the nursing care culture.

## METHODS

### Ethical aspects

This study uses the historical method with documentary sources from nursing manuals dating from 1875 to 1928 for the construction of the profession’s historiography, with emphasis on the care provided. Therefore, there was no need for submission to an Ethics Committee for Research^(15-17)^.

### Type of study

Historical study, in the cultural perspective^(18)^, using the technique of documentary analysis^(19)^.

### Data source

The documents used in the research were nursing manuals in Portuguese, French, English, and Spanish available in the virtual collection of the Gallica website, from the National Library of France; the Library of Ghent University, Belgium; the Library of Johnson & Wales University, Charlotte Campus, United States; the United States National Library of Medicine, United States, and the SOPHIA system, from the Central Library of the Federal University of Rio de Janeiro Guilherme Figueiredo.

### Data collection and organization

The temporal delimitation was from 1875 to 1928, the period of publication of the studied manuals. The justification for the initial period was the English publication of the Manual for Hospital Nurses and Others Engaged in Attending on the Sick^(20)^ and the final period with the Brazilian work entitled “*Livro do Enfermeiro e da Enfermeira*”^(21)^.

For data collection, an instrument with filled spaces was used, namely: title of the work; year of publication; language; authorship; location; formation; and synthesis of the addressed theme of interest to the study object.

As inclusion criteria, the manuals in Portuguese (Brazil), French, Spanish, and English were considered, and those that did not mention care in orthopedics were excluded, in the proposed temporal delimitation. For the search, we applied the established criteria and the terms related to orthopedics: fracture, dislocation, sprain, and immobilization, in the proposed languages.

### Data analysis

Through the collection of information, we organized the data in tables for the unfolding of the discussion, limitations, contributions, and final considerations. The data were triangulated with each other^(22)^ and/or with adherence literature in the discussion for the construction of the historical narrative with its versions and interpretations^(23)^.

## RESULTS

The search resulted in a set of 22 manuals. However, after applying the established criteria, the corpus of analysis was comprised of 12 works. Chart 1 presents a summary of the nursing manuals with emphasis on orthopedic care.

**Chart 1:** Summary of Nursing Manuals with Emphasis on Orthopedic Care (1875-1928)

Year	Title	Author/Profession	Summary
1875	Manual for Hospital Nurses and Others Engaged in Attending on the Sick^(20)^	Edward J. Domville / Physician	The manual provides knowledge about fractures and dislocations, emphasizing the importance of immediately immobilizing the affected limb with splints to avoid complications from the injury.
1878	*Manuel pratique de la garde-malade et de l’infirmière* ^(24)^	Dr.Bourneville / Physician	In this text, orthopedic care is represented through fracture devices that were designed to immobilize the affected area. Additionally, the text states that nurses were not responsible for performing these procedures, but rather for identifying them to communicate with the doctors.
1891	*Manuel Théorique Et Pratique De Bandages* ^(25)^	Edmond Morim / Physician	The manual covers the classification, purpose, and technique for applying bandages.
1900	A handbook of nursing^(26)^	M.N. Oxford / Sister of Charity	The manual reports that nurses were not authorized to perform care for fractures. They should be able to recognize and classify fractures. Additionally, the work covers first aid, precautions during transportation, bed arrangement, and patient accommodation in order to avoid further injury.
1901	*École de L’infirmier et du Brancardier Militaires* ^(27)^	French Ministry of War	The work discusses how to rescue and transport the injured with orthopedic problems without aggravating their condition, as well as providing readers with lessons in first aid in case of fractures.
1914	Practical nursing - a text-book for nurses^(28)^	Anna Caroline Maxwell e Amy Elizabeth Pope / Nurses	This manual’s approach is directed towards the use and types of bandages, fixations, and splints, as well as their purposes and application. It also presents plaster casts. Distinguishing between dislocations, fractures, sprains, and bruises as emergencies is emphasized. Alternative methods for relieving pain and symptoms such as cold and hot compresses in the case of bruises and massages in the case of sprains are highlighted.
1915	*Marine nationale. Manuel du marin infirmier* ^(29)^	National Navy of the French Republic	The manual conceptualizes fracture, its classifications, how to recognize it, the forms of care, and how to transport the injured without causing further harm. Among the care measures, it highlights the containment or immobilization with the application of splints and other devices.
1915	*Manuel de l’infirmière: petite chirurgie et soins d’urgence* ^(30)^	Camille Fromaget / Physician	The work presents definitions and care for injuries such as sprains, dislocations, and fractures. First aid is addressed, from immobilization and transportation to post-injury care. It differentiates types of immobilizations and their respective purposes, such as pain relief and preventing further damage.
1915	*Licções do curso pratico para as Damas enfermeiras voluntarias, de accordo com o programma approvada - PART II* ^(31)^	Brazilian Red Cross Society	The manual provides guidance for nurses on how to care for patients with dislocations and fractures, including pain relief, hemostasis, preparation of accommodations, changing bed linens and dressing wounds.
1920	*Curso de Enfermeiros* ^(32)^	Adolpho Possollo / Physician	The book reports that orthopedic care was directed at patients with bandages and casts. It emphasizes the importance of ensuring stability and immobilization of the fractured area, and presents devices such as gutters and other orthopedic appliances.
1925	*Le Livre de L’Infirmière* ^(33)^	M.N. Oxford / Sister of Charity. Translation: Léonie Chaptal / Nurse	The concept of fracture is presented, as well as its causes, types, and other details. The nurse is taught about general principles and precautions for dealing with orthopedic injuries, especially in relieving pain.
1928	*Livro do Enfermeiro e da Enfermeira - para uso dos que se destinam à profissão de enfermagem e das pessoas que cuidam de doentes* ^(21)^	Getúlio dos Santos / Physician	The author mentions the nurse as an assistant to the doctor in preparing the immobilization of the injured limb, for example. It explains that the nurse should differentiate between dislocation and fracture, know how to handle casts, perform wound cleaning, and observe for the presence of edema or infection after applying the appliances. The book also provides guidance on first aid.

In summary, the authors of the analyzed manuals include 6 physicians, 3 nurses, 3 institutions, and 1 Sister of Charity. The manuals were published in 6 in French, 3 in English, and 3 in Portuguese. However, it was not possible to find manuals in the Spanish language based on the criteria adopted by the methodology.

## DISCUSSION

The method proposed by the manuals meets two principles: order and distancing from banalized reality. They lead to domestication for the sake of the practice to be internalized in gestures and behaviors. Therefore, it is pedagogically simplistic, as it does not enable learning to think but to reproduce, as it produces generalizations and uniform thoughts, which has the effect of mechanistic behavior^(34)^.

Therefore, it is possible to identify the prescription of care in the manuals, in which nurses should be taught to recognize fractures, sprains, or dislocations through signs and symptoms, in order to minimize the risk of complications in proper handling. This implies that they should have standardized conduct for the successful care of patients as one of the ways of inculcating the culture of care, considering the microbiology knowledge of Louis Pasteur (1822-1895).

The identified manuals were mainly directed at nurses and authored by physicians in the three languages. Each of them, while writing, informed their intentions in forming the culture of care. This, delimited by the works, can be interpreted as a dynamic in favor of the professionalization of nursing, which began in France in 1877, considering that before that date, care was provided by religious women, emerging the movement of inserting lay nurses under the domination of medicine^(34)^.

It should be noted that two manuals are authored by nurses. The first, in English (1914)^(28)^, and the second, in French (1925)^(33)^. Of the two, the one entitled *Le Livre de L’Infirmière* ^(33)^, authored by Léonie Chaptal (translation of A Handbook of Nursing^(26)^ by M.N. OXFORD), is one of the protagonists in defense of nursing distinct from the proposal by Désiré-Magloire Bourneville and in favor of what the English nurse Florence Nightingale advocated.

In other words, Léonie Chaptal believed that the nurse should know the patient, their environment, care for their health conditions and prevent further damage without any disagreement between the nurse and the physician. Moreover, the nurse should execute what medical science decides, being an agent of the physician and the institution at the service of the patient^(34)^, and the successful implementation of modern nursing in France over the years^(1)^.

Understanding the logic that points to the results of manuals in English and French languages is evidence of the culture of care that nurses should execute when “learning” through them for their practices. This implies distinguishing French manuals in favor of the liberation struggle against religious power for domination in prescribing care by physicians. The aspirational model is the one that should be played by nurses through the teachings of Florence Nightingale.

In the delimitation of the culture of orthopedic care, manuals refer to first aid. The subject brings argumentation for conflicts in warfare when injured people were targeted and needed to be transported due to locomotor system damage.

Historically, first aid originated from the period of the great wars in the 18th century, the Napoleonic period, when battlefield casualties were transported in animal-drawn carts to be cared for and treated. Thus, the surgeon and military chief Dominique Larrey began to serve in the battlefield in 1792 with the aim of preventing complications for the lives of those affected. This resulted, over the years, in the organization when it was associated with the International Red Cross for its proposal for appropriate training for care and rescue^(35)^.

In the manuals researched to identify the culture of orthopedic care, four works were found that refer to first aid^(21,26-27,30)^. Of these, one is in English^(26)^, two in French^(27,30)^, and one in Portuguese (Brazil)^(21)^, with two having direct articulation with the trajectory of first aid in French^(21)^ and Portuguese^(27)^. Two manuals can be articulated since the authors have a military background.

The above paragraphs aim to frame orthopedic care to be mentioned from this moment on. To begin, we bring the culture of applying compresses to relieve pain. According to the records, they could be cold, soaked in alcohol or camphor, and applied to the affected limb in cases of edema or deformation, as well as indicating the combat of inflammation.

The application of compresses (hot/cold) dates back to 2500 BC. It was used by the Egyptian, Greek, and Roman people as one of the methods for analgesia and anti-inflammatory purposes. This practice has crossed centuries and with time, what was done at home was inserted as a culture of care^(36)^. The practice of applying water compresses with or without substances produced effects. Nowadays, they remain in teaching and as a culturalized practice in care, but with a lower status than in the past.

Another element identified for orthopedic care was the application of iodine tincture to disinfect exposed fractures in dressings. Historically, the French chemist Jean Baptiste André Dumas (1800-1884) sought to produce an iodine-based medicine to be orally administered for the treatment of patients with goiter caused by iodine deficiency in the human body, but he failed. Thus, the solution was the preparation of an alcoholic solution of iodine and potassium iodide, which gave rise to iodine tincture (1819). Scientific evidence emerged as a germicidal action (1873), when the French bacteriologist Casimir Davaine (1812-1882) demonstrated that it inhibited the proliferation of the Bacillus anthracis, the cause of infectious carbuncle (anthrax)^(37)^. This pathogen causes gangrene. Therefore, the indication at that time was the application of iodine tincture taught to nurses as prevention of infection, in addition to the hygiene of the exposed fracture.

Bandages were another care for orthopedic problems. Nurses could perform them, provided they had knowledge of the technique presented in texts and images. The bandaging technique is a tradition from Egypt destined for the mummification of people and animals^(38)^. A vestige left by the past, in ancient Greece, in a piece known as the cup of Sosia, makes it possible to identify the use of bandages in wrapping. It is a scene of Achilles wrapping the injured arm of his friend Patroclus in the spike typology. According to Greek mythology, he possessed medical knowledge transmitted by the centaur Orion, a doctor^(39)^.

In the search for vestiges of bandages in the culture of orthopedic care, we realize how traditional this technique is. Moreover, it is possible to relate care for the dead body in Egypt, Greek art, and care for people with problems in their limbs.

Immobilization is one of the elements that draws attention in the culture of orthopedic care, as observed in the consulted works, considering the variety of materials used. This resulted in the organization of four mosaic images below.

**Figure 1:**
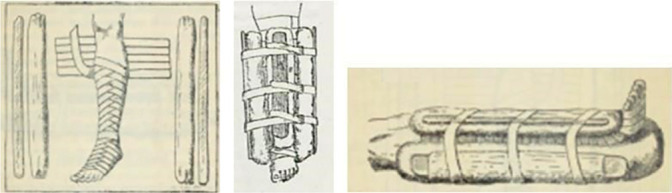
Appareil de Scultet ^**(25,29)**^

In Figure 1, we present the *Appareil de Scultet*. This device is made with a sheet stretched behind the limb, type barbela, composed of six straps fixed on its sides, which embrace the wrapped limb with the sides padded on both sides with wooden splints, as described in the works^(25,29)^.

Despite the distant dates (1891 and 1915), both are in the French language. This implies evidence that they were applied in healthcare institutions in France, and we infer those other hospitals in Europe applied the same type of immobilization. As we can identify in the presented images from distinct works, they show care for the lower limbs.

**Figure 2:**
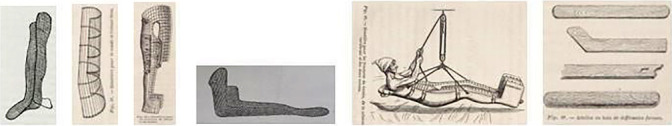
Gutters, splints, and braces^(21,24-25)^

In Figure 2, we present splints, braces, and gutters. As we can identify in the six mosaic images, the first four images are the conveyed gutters, isolated or not^(21,24-25)^, and the fourth and fifth image from left to right are from the same work^(24)^.

The gutter is a translation of the French word “*Gouttières*” and is a wired gutter in the shape of the posterior part of the upper and lower limb. As we can identify, they are mostly from works in French, but it is noteworthy that it appears in a work from 1928 in Brazilian Portuguese. Here, we highlight that the piece is in the collection of the Alfredo Pinto Nursing School, and we infer that it may be an artifact from the past to teach how to use it on orthopedic patients.

The fifth image in the mosaic in Figure 2 is called “*Gouttière pour les fractures du bassin, de la colonne vertébrale et des deux cuisses*”^(24)^, which translates as “gutter for fractures of the pelvis, spine, and lower limbs.” As can be observed, it depicts a man restrained by a crank with articulated pulleys attached to a gutter.

The sixth image consists of four straight and curved wooden splints^(24)^. It was intended for use on the upper and lower limbs. We infer that its application was padded to prevent further damage.

The next immobilization modality is the plaster cast applied in orthopedic care. Its application was intended for adults and children in the visual representations observed in the consulted works.

**Figure 3:**
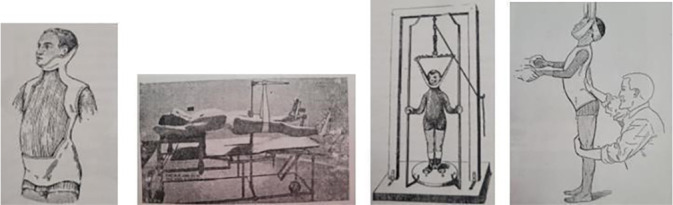
Plaster cast apparatus^(32)^

Figure 3 consists of four images, all from the book entitled “*Curso de Enfermeiros*” (1920)^(32)^.

The first image, from right to left, depicts an open window, called the Minerva apparatus. It is a representation of a man with a piece removed from the plaster cast apparatus, leaving the desired exposed body part uncovered.

The second image is considered the Kny-Sheerer orthopedic table. It is an imagery representation of a man restrained by the legs while the trunk is supported on the table. This was used for applying the apparatus on the leg and lower limbs.

Image three in Figure 3 is the suspension apparatus for applying the plaster jacket. In the image, we observe a representation of a child restrained by the head and suspended by a rope or similar material for applying the plaster on the chest.

Finally, the fourth image was used in the placement of the plaster apparatus on the trunk. For better positioning and utilization for plaster application. The figure depicts a representation of a child restrained by the head with a band to help the person holding the child’s hands while another adjusts it by pressing the spine forward with one hand and positioning the knee from behind to the front with the other.

**Figure 4:**
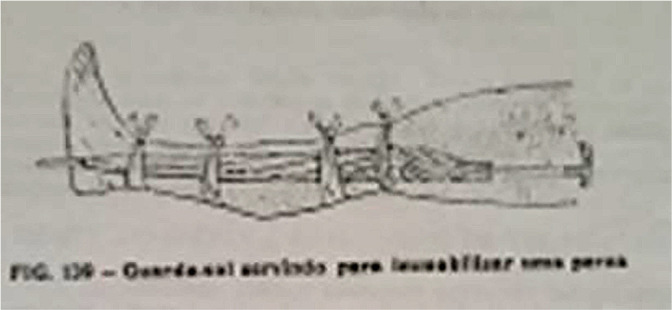
First aid assistance to a person suspected of having a fracture^(21)^

Figure 4 shows fixation for immobilization in first aid care. The representation is to illustrate the text of the work “*Livro do Enfermeiro e da Enfermeira - para uso dos que se destinam à profissão de enfermagem e das pessoas que cuidam de doentes*”^(21)^.

In Figure 4, immobilization is performed using an umbrella. We observe the representation of the lower limb with the aforementioned device and four ties as a fixation strategy. This immobilization is of interest, as it points to direct adherence to the author’s institution of action - Getulio dos Santos in the Brazilian Red Cross - considering the purposes of acting in peace and war. Thus, we infer that, even though the author’s book is not exclusively aimed at first aid, there are strong indications of institutional belonging to the point of inserting one of the immobilization strategies in situations outside the hospital setting.

It should be noted that, in this study, we had the opportunity to show some images of immobilization. However, this does not mean that the other consulted works, without illustrations, lacked the presentation of immobilization types. We emphasize that the highlighting of books with images was due to our understanding that they show more than words can represent.

Through the teachings, concepts, and strategies of identification and management of signs of edema and hemorrhages, for example, learned by the aspiring nurses, they needed to act. This action was limited but relevant, especially for alert signs of interventions.

In other words, by identifying complications, they communicated them to the doctor, and sometimes, it was necessary for them to act to prevent more serious complications until the doctor arrived. Thus, metaphorically, they were the eyes and arms of doctors in their absence to act in emergency cases.

### Study limitations

We understand that more works could have been included, but we believe that not all of them are digitized for public consultation, especially foreign ones, which could lead to other versions and interpretations of the culture of orthopedic care.

### Contributions to Nursing and Health Practice

We understand that the contributions include presenting the authorship of foreign nurses in the proposed delimitation, considering that in Brazil, this occurred after the 1930s. Additionally, the research may contribute to the consultation of the Federal Nursing Council on specialization in Orthopedic Nursing, by highlighting a practice that constitutes an element of the culture of care.

## CONCLUSIONS

The investigation fulfilled its purpose of discussing the contents of manuals, with an emphasis on orthopedics, for the development of the culture of care, by revealing the analyzed and discussed works. This implied highlighting the old practice of orthopedic nursing and its relevant effect in assisting those with motor needs caused by fractures or similar conditions.

Visiting the past through these works means reflecting on how our predecessors were prepared to get where we are today. Understanding the process of professionalization through struggles and achievements, for the occupation of sociocultural spaces, and the role of nurses, even if limited at various moments, emphasizes the need for investment in their performance and a high level of observation of warning signs so that doctors could act, with some exceptions.

Both in this investigation and in others, the documentary source produced over the years proves important for the present. They bring evidence, traces, and information about how the events occurred and who were the characters involved in the culture of care. This reveals possibilities of constructing plausible narratives for the present time. Therefore, they are investigative windows that open up for us to advance more and more in the political, economic, sanitary, and sociocultural fields for global nursing.
